# Inhibition of microRNA-21 upregulates the expression of programmed cell death 4 and phosphatase tensin homologue in the A431 squamous cell carcinoma cell line

**DOI:** 10.3892/ol.2014.2066

**Published:** 2014-04-15

**Authors:** XIAOHONG LI, KAI HUANG, JIANBIN YU

**Affiliations:** 1 Department of Dermatology, The First Affiliated Hospital, Zhengzhou University, Zhengzhou, Henan 450052, P.R. China; 2 Department of Oncology, The First Affiliated Hospital, Zhengzhou University, Zhengzhou, Henan 450052, P.R. China

**Keywords:** carcinoma, squamous cell, microRNA-21, programmed cell death 4, phosphatase tensin homologue, apoptosis

## Abstract

microRNA-21 (miRNA/miR-21) is a well-known oncogenic miRNA that is overexpressed in various carcinomas. The tumor suppressor genes, programmed cell death 4 (PDCD4) and phosphatase tensin homologue (PTEN), which target miR-21, are underexpressed in several types of cancer. However, the expression of miR-21 and its target genes, PDCD4 and PTEN, has not yet been reported in skin squamous cell carcinoma (SCC). In the present study, anti-miR-21 was transfected into the A431 cell line, and the expression of miR-21, PDCD4 and PTEN and the level of cell apoptosis were detected by quantitative polymerase chain reaction, immunocytochemistry and western blotting, and terminal deoxynucleotidyl transferase-mediated dUTP nick end labeling, respectively. The expression levels of PDCD4 and PTEN in the A431 cell line transfected with anti-miR-21 were significantly increased (P<0.05) and the apoptotic ratio was significantly increased (P<0.05). The data indicate that miR-21 may play an oncogenic role in the cellular processes of SCC and represent a novel target for effective therapies.

## Introduction

Squamous cell carcinoma (SCC) is the second most common type of non-melanoma cancer of the skin. Although there are a number of associated risk factors, the etiology of this cancer has not yet been determined. microRNA-21 (miRNA/miR-21) is overexpressed in several types of solid tumors, including esophageal (1), stomach (2), colorectal (3), prostate (4), pancreatic (5), lung (6) and head and neck (7) cancers. It has been reported that the downregulation of miR-21 suppresses tumor growth and invasion in breast, glioma and colon cancer cells. Furthermore, the inhibition of miR-21 can regulate the expression of phosphatase tensin homologue (PTEN) and programmed cell death 4 (PDCD4) in cancer cells. However, the biological roles of miR-21 in SCC of the skin remain poorly understood and require further study. To the best of our knowledge, no previous studies have investigated the role of miR-21 in the A431 cell line. The present study was the first to examine the expression of PDCD4, PTEN and cell apoptosis in A431 cells transfected with anti-miR-21.

The findings of the current study demonstrate that miR-21 plays an oncogenic role in the process of SCC and may serve as a target for effective therapy of SCC of skin.

## Material and methods

### Cell culture

The A431 cells of human cutaneous SCC were kindly provided by the Cell Resource Center of the Institute of Basic Sciences, Chinese Academy of Medical Sciences (Tianjin, China). The cells were cultured in Dulbecco’s modified Eagle’s medium containing 10% fetal bovine serum (Gibco-BRL, Carlsbad, CA, USA) and incubated in a humidified atmosphere of 5% CO_2_ at 37°C. The cells were divided into three groups as follows: i) untreated A431 cells; ii) A431 cells transfected with an unrelated fragment (negative control); and iii) A431 cells transfected with antisense oligonucleotide (ASO)-miR-21.

### Quantitative polymerase chain reaction (qPCR)

For the A431 cells, total RNA was isolated using TRIzol reagent (Invitrogen Life Technologies, Carlsbad, CA, USA) according to the manufacturer’s instructions. Total RNA (~200 ng) was reverse transcribed using gene-specific reverse transcription primers from the TaqMan microRNA assays (Applied Biosystems, Foster City, CA, USA) and the TaqMan microRNA Reverse Transcription kit (Takara Bio, Inc., Shiga, Japan) according to the manufacturer’s instructions. qPCR was performed on the iQ5 Multicolor Real-time Detection System (Bio-Rad, Hercules, CA, USA) under the following conditions: initial denaturation for 3 min at 95°C, followed by 40 cycles for denaturation for 30 sec at 95°C, combined annealing for 30 sec at 56°C and primer extension for 30 sec at 72°C. To estimate the expression of miR-21, the Ct values were normalized using 18S rRNA as an internal control. The relative miRNA expression level was calculated using the 2^−ΔΔCt^ method. The primer for miR-21 detection was 5′-TGCGGTAGCTTATCAGACTGATG-3′, and the heat shock RNA-U6 primer was 5′-TGCGGGTGCTCGCTTCGG CAGC-3′.

### Transfection of ASO-miR-21

ASOs of human miR-21 were transfected by Lipofectamine™ 2000 reagent (Invitrogen Life Technologies). The primer sequence of ASO was 5′-TCAACA TCAGTCTGATAAGCTA-3′.

### Immunocytochemistry

PCDC4 and PTEN protein expression was detected by immunocytochemistry of streptavidin-perosidase. Following culture, the cells were grown on microscope slides and sections were deparaffinized, heated in a microwave in 0.01 M sodium citrate buffer for antigen retrieval, treated with 3% H_2_O_2_ for 10 min and rinsed in H_2_O and phosphate-buffered saline (PBS). The sections were blocked in 5% goat serum in PBS, followed by incubation with the anti-PDCD4 and anti-PTEN antibodies (both Tianjin Saier Biotechology Co., Ltd., Tianjin, China). Signals were detected with 3,3′-diaminobenzidine substrate (ZSGB-Bio, Inc., Beijing, China). PDCD4 and PTEN protein expression was evaluated by integrated optical density.

### Western blot analysis

The expression levels of downstream targets of human miR-21 were determined by western blot analysis. Protein from the A431 cells was extracted using radioimmunoprecipitation assay lysis buffer (Tianjin Saier Biotechology Co., Ltd.). Samples were resolved using SDS-PAGE on a 10% Tris-HCl gel and transferred to a nitrocellulose filter membrane (Millipore, Billerica, MA, USA). The membrane was probed with specific antibodies and target proteins, including GAPDH, rabbit anti-PDCD4 and rabbit anti-PTEN. Horseradish peroxidase (HRP)-conjugated secondary antibodies (goat anti-rabbit HRP) and luminal reagent were used to detect chemiluminescence. Blots were subsequently exposed to X-ray film and developed. Bands were digitally scanned and analyzed with Labworks 4.0 system (UVP, LLC, Upland, CA, USA). Western blotting of GAPDH on the same membrane was used as a loading control. The average pixel densities and band sizes in the control bands were used to normalize band density and the size of the target proteins. Target bands from the cells were directly compared.

### Apoptosis assay

The three groups of cells were washed twice with 10 mM cold PBS and resuspended in 1× binding buffer at a concentration of 1×10^6^ cells/ml. The cells were stained with 4′,6-diamidino-2-phenylindole (DAPI) and terminal deoxynucleotidyl transferase-mediated dUTP nick end labeling (TUNEL), using the FragEL™ DNA Fragmentation Detection kit (Merck KGaA, Darmstadt, Germany). The experiments were repeated at least three times. DAPI stained the nucleus of all the cells, while fluorescein stained the nucleus of the apoptotic cells. The ratio of apoptotic cell to all the cells was used to evaluate the level of cell apoptosis.

### Statistical analysis

Data are expressed as the mean ± standard deviation of three independent cell groups. The differences between groups were assessed by an unpaired two-tailed Student’s t-test. P<0.05 was considered to indicate a statistically significant difference.

## Results

### ASO-miR-21 downregulates miR-21 in A431 cells

**Figure 1:**
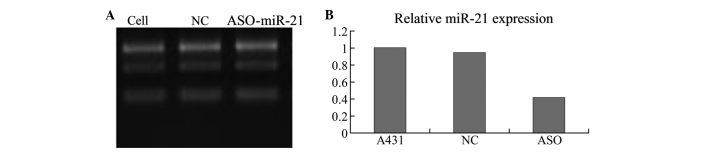
(A) Quantitative polymerase chain reaction (qPCR) result demonstrating mRNA expression of miR-21 in A431 cells, cells transfected with an unrelated fragment of control (NC) and cells transfected with ASO-miR-21. (B) The relative expression level of miR-21 in the A431 cells was significantly decreased by ASO-miR-21 compared with the control (P<0.05). NC, normal contol; ASO, antisense oligonucleotide; miR, microRNA.

qPCR analysis demonstrated that the three groups of cells had different expression levels of miR-21 (F=107.24, P<0.05). miR-21 was expressed at a significantly higher level in the A431 cells and cells transfected with an unrelated fragment of control compared with the cells transfected with ASO-miR-21 (P<0.05). However, similar levels of miR-21 expression were found between the control A431 cells and the cells without treatment (P>0.05). Thus demonstrating that ASO-miR-21 downregulates miR-21 in A431 cells (Fig. 1).

### ASO-miR-21 upregulates the expression of PDCD4 and PTEN in A431 cells

**Figure 2:**
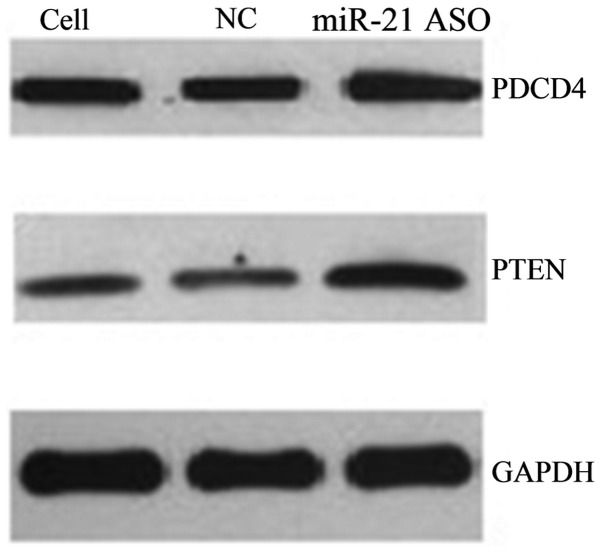
Western blot analysis of PDCD4 and PTEN expression in A431 cells. PDCD4 and PTEN expression in the A431 cells was evidently increased by ASO-miR-21 compared with the control (P<0.05). PDCD4, programmed cell death 4; PTEN, phosphatase tensin homogue; ASO, antisense oligonucleotide; miR, microRNA; NC, negative control.

**Figure 3:**
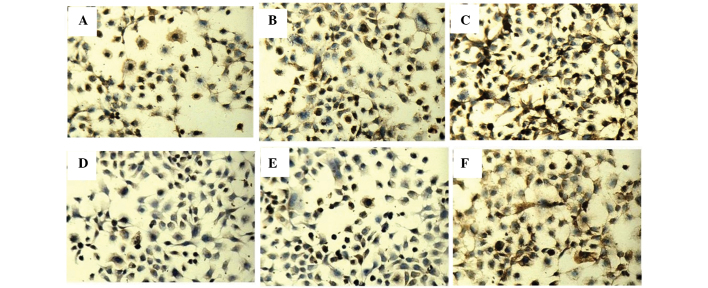
Expression of PDCD4 in the (A) A431 cells, (B) cells transfected with an unrelated fragment of control and (C) cells transfected with ASO-miR-21. Expression of PDCD4 in the (D) A431 cells, (E) cells transfected with an unrelated fragment of control and (F) cells transfected with ASO-miR-21. The expression of PDCD4 and PTEN in the A431 cells was evidently increased by ASO-miR-21 compared with the control, as detected by immunocytochemistry (P<0.05). PDCD4, programmed cell death 4; PTEN, phosphatase tensin homologue; ASO, antisense oligonucleotide; miR, microRNA.

Western blot analysis showed that the three groups of cells had different expression levels of PDCD4 (F=11,941.13, P<0.05) and PTEN (F=83,249.64, P<0.05). Following ASO-miR-21 transfection, the expression of PDCD4 and PTEN in the A431 cells was evidently increased compared with the control group (Figs. 2 and 3), while similar levels of PDCD4 and PTEN expression were found between the control group and the cells without any treatment. In addition, to determine the effects of miR-21 on PDCD4 and PTEN expression, the protein levels of PDCD4 and PTEN were detected by immunocytochemistry and western blotting. The positive staining of PDCD4 and PTEN was localized in the cytoplasm and the nucleus (Fig. 3). Immunocytochemistry revealed that the three groups of cells had different expression levels of PDCD4 (F=50.12, P<0.05) and PTEN (F=576.54, P<0.05).

### Downregulation of miR-21 induces apoptosis of A431 cells

**Figure 4:**
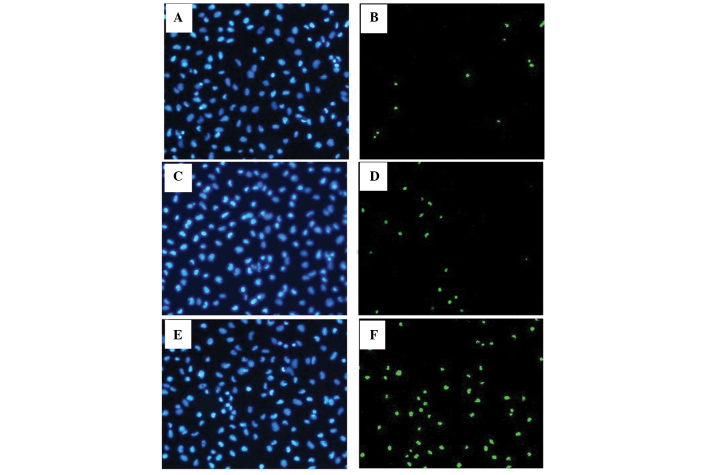
Apoptosis in A431 cells stained with (A) DAPI and (B) TUNEL, cells transfected with an unrelated fragment of control stained with (C) DAPI and (D) TUNEL, and cells transfected with ASO-miR-21 stained with (E) DAPI and (F) TUNEL. The apoptotic ratio in the A431 cells was evidently increased by ASO-miR-21 transfection conpared with the control group (P<0.05). DAPI, 4′, 6-diamidino-2-phenylindole; TUNEL, terminal deoxynucleotidyl transferase-mediated dUTP nick end labeling; ASO, antisense oligonucleotide; miR, microRNA.

To determine the effects of miR-21 on apoptosis, cell apoptosis was detected by TUNEL assay and the apoptotic ratio was used to evaluate the level of apoptosis in the cells. The three groups of cells had different apoptotic ratios (F=201.79, P<0.05). Following ASO-miR-21 transfection, the apoptotic ratio in the A431 cells was evidently increased compared with the control group (Fig. 4), while similar apoptotic ratios were found between the control A431 cells and the cells without any treatment.

## Discussion

SCC is one of the most common types of skin cancer in dermatology, and there are numerous risk factors associated with SCC of the skin, including ultraviolet-B radiation, radiation therapy, previous burns, exposure to arsenic and coal tar, human papilloma virus infection, inflammatory lesions and ulcers of long standing (8). miR-21 is overexpressed in several types of cancers and induces the invasion, intravasion and metastasis of cancer (9). PDCD4 and PTEN are the target genes of miR-21. In the present study, we hypothesized that miR-21 downregulates the expression of PDCD4 and PTEN in A431 cells, and that the inhibition of miR-21 subsequently increases PDCD4 and PTEN expression and suppresses tumor cell growth. The results showed that miR-21 affected the expression of PDCD4 and PTEN and the apoptosis of the A431 cells.

PDCD4 suppresses several targets that regulate translation and cell proliferation, and has been indicated to be involved in tissue invasion and proliferation. In a mouse cancer model, PDCD4 suppressed benign and malignant skin tumor formation and progression (10). In a study of colorectal cancer, PDCD4 mRNA levels were negatively regulated by miR-21 at each stage of cancer (11). PTEN is a phosphatase that maintains low levels of phosphatidylinositol 3,4,5-triphosphate (PIP-3) by conversion to PIP-2. When PTEN fails to maintain this homeostasis, PIP-3 levels increase and activate the protein kinase B (Akt) pathway. Activation of the Akt pathway has several effects, including the promotion of cell growth and proliferation and the inhibition of apoptosis (12,13). Meng *et al* reported that the aberrant expression of miR-21 contributed to hepatocellular carcinoma growth and spread by modulating PTEN expression and PTEN-dependent pathways involved in mediating the cell growth, migration and invasion of cancer cells (14). Ming and He reported that PTEN negatively regulates the oncogenic phosphatidylinositol 3-kinase/Akt signaling pathway and showed that PTEN is a critical tumor suppressor for skin cancer in humans and in mice (15). A previous study of gastric cancer indicated that miR-21 inhibition may upregulate the PTEN expression level, and that the downregulation of miR-21 exhibits a stronger inhibitory effect on the biological behavior of cancer cells (16).

In the present study, ASO-miR-21 was efficiently transfected into the A431 cells resulting in a marked downregulation of miR-21 *in vitro*. Previous studies have shown that transfection of anti-miR-21 can downregulate miR-21 expression of oral SCC (17) and laryngeal SCC (18). In the present study, immunocytochemistry and western blot analysis revealed a significant increase in the expression of PDCD4 and PTEN in the A431 cell line transfected with anti-miR-21. Previous studies have shown that the inhibition of miR-21 in cancer cells increases PTEN and PDCD4 protein levels in HeLa and MCF-7/ADR cells (19,20). Furthermore, in the present study, the TUNEL assay showed a significant increase in the apoptotic ratio in the A431 cell line transfected with anti-miR-21. miR-21 has also been found to be upregulated in laryngeal carcinoma tissues, and the knockdown of miR-21 by a specific ASO inhibited the proliferation potential of Hep-2 cells (21).

In conclusion, the present study demonstrated that miR-21 downregulates the expression of PDCD4 and PTEN, and that the inhibition of miR-21 suppresses tumor growth and invasion. Considering that PDCD4 and PTEN function as tumor suppressor genes and are the target genes of miR-21, ASO-miR-21 may have potential applications as a therapeutic target. Understanding the complex regulation of miR-21 with regard to its target gene expression in SCC may be valuable for exploring potential therapeutic methods for SCC, and gene therapy targeting miR-21 should be further investigated as a potential alternative strategy for SCC therapy.
